# Identification and Characterization of MYB-bHLH-WD40 Regulatory Complex Members Controlling Anthocyanidin Biosynthesis in Blueberry Fruits Development

**DOI:** 10.3390/genes10070496

**Published:** 2019-06-28

**Authors:** Mengran Zhao, Jian Li, Ling Zhu, Pan Chang, Lingli Li, Lingyun Zhang

**Affiliations:** 1 College of Forestry, Northwest A&F University, Yangling 712100, China; 2 Shaanxi Province Key Laboratory of Economic Plant Resources Development and Utilization, Yangling 712100, China; 3 Key Laboratory of Forest Silviculture and Conservation of the Ministry of Education, Beijing Forestry University, Beijing 100083, China

**Keywords:** *Vaccinium corymbosum*, transcription factors, flavonoids, fruit ripening

## Abstract

Anthocyanins is the main representative of flavonoids in blueberry fruits. The anthocyanins biosynthetic pathway has been extensively studied in numerous model plants and fruit crops at biochemical, genetic, and molecular levels. However, the mechanisms by which the MYB transcription factor/basic helix-loop-helix (bHLH) domain protein/WD-repeat (MYB-bHLH-WD40) complexes regulate anthocyanin biosynthesis in blueberry is still limited. In the present study, we identified 11 *MYB*, 7 *bHLH*, and 6 *WD40* genes in blueberry fruits, using amino acid sequences of homologous MYB-bHLH-WD40 complexes in *Arabidopsis*, apple, grape, and strawberry. To understand these mechanisms, the expression patterns of *MYB-bHLH-WD40* genes were examined and validated using differentially expressed gene (DEG) analysis and quantitative real-time reverse transcription PCR (qRT-PCR), respectively. The expression patterns of *MYB-bHLH-WD40* genes positively correlated with anthocyanin accumulation and color development in blueberry fruits. Consistent with the effects of other transcriptional regulators, the VcMYBL1::GFP, VcbHLH1::GFP, and VcWDL2::GFP fusion proteins were only observed in the nucleus. The protein-protein interactions (PPIs) and bimolecular fluorescence complementation (BiFC) assay suggested a possible link between VcbHLHL1 and VcMYBL1. Finally, a model was proposed and discussed for how the expression of the MYB-bHLH-WD40 complexes can promote anthocyanin biosynthesis in blueberry fruits. To our knowledge, this study was the first to evaluate MYB-bHLH-WD40 complexes in blueberry fruits, and it provides a foundation to dissect the function of the mechanism.

## 1. Introduction

Blueberries (*Vaccinium corymbosum*), also known as lingonberries, are perennial flowering shrubs with indigo-colored berries. They are classified in the section *Cyanococcus* within the genus *Vaccinium* of the Ericaceae [1,2]. The popularity of the blueberry as an economically important small fruit crop is mainly the result of its unique flavor, rich nutrients, and prevention of multiple diseases. These quality traits are largely determined by the anthocyanins. Anthocyanins as an important class of flavonoids in plant polyphenols, not only determine the color of the fruit [3,4], but also the main source of antioxidant activity in the blueberry fruit [5,6,7]. The anthocyanins contained in the blueberry fruit have a certain effect on improving vision, delaying memory decline, reducing the incidence of cardiovascular and cerebrovascular diseases and cancer, and resisting oxidation. They are listed as one of the top five human health foods by the Food and Agriculture Organization [8,9].

Anthocyanins are the main representatives of flavonoids in the blueberry fruit. The flavonoid biosynthetic pathway has been extensively studied in numerous model plant and fruit crops at biochemical, genetic, and molecular levels [10,11,12,13,14]. Several genes encoding the biosynthetic enzymes and transcription factors (TF) of this pathway have been extensively studied in maize, *Arabidopsis*, petunia, tobacco, and fruit crops, such as grape, apple, strawberry, and others [15,16,17,18,19,20,21,22,23,24,25]. In maize, the transcriptional regulation of anthocyanins biosynthesis is through the MYB transcription factor/basic helix-loop-helix (bHLH) domain protein/WD-repeat (MYB-bHLH-WD40) protein complex activated by the structural genes, in which the bHLH member plays a central role and interacts with the MYB and WD40 TF protein [26,27,28]. In *Arabidopsis*, the anthocyanin biosynthetic gene is also regulated by the MYB-bHLH-WD40 complex, although it can be activated by the R2R3-MYB transcription factor alone at an early stage [29].

The anthocyanin biosynthetic pathway is also described in fruit crops. The anthocyanin synthesis models of strawberry and apple are similar to that of *Arabidopsis*. The strawberry MYB-bHLH-WD40 regulatory complexes (FaMYB9, FaMYB11, FabHLH3, and FaTTG1) show homology to AtTT2, AtTT8, and AtTTG1, and the abundance of anthocyanins and procyanidins in apples are regulated by the WD40 protein MdTTG1, though it only interacts with bHLH. In grape, the MYB-bHLH-WD40 complexes are involved in the transcriptional regulation, via the *VvMYB5B*, *VvMYBCS1*, *VvMYC1*, *VvMYCA1*, and *VvWD* genes [30,31,32,33,34]. However, there is still limited information available on the transcriptional regulation of the anthocyanin biosynthesis pathway via the MYB-bHLH-WD40 protein complexes in blueberry fruits.

To gain more insight into the regulation of anthocyanin biosynthesis during blueberry fruit and color development, the transcriptome sequencing data released with our previous publication [35] including green, pink, and blue fruit developments were further explored. In the present study, 11 *MYB*, 7 *bHLH*, and 6 *WD40* genes were obtained from blueberry fruits, using amino acid sequences of homologous MYB-bHLH-WD40 complexes in *Arabidopsis*, apple, grape, and strawberry. The *MYB-bHLH-WD40* gene expression patterns positively correlated with anthocyanin accumulation in the blueberry fruit and color development. Consistent with their roles as the transcriptional regulator, VcMYBL1::GFP, VcbHLH1::GFP, and VcWDL2::GFP fusion proteins were observed only in the nucleus. The protein–protein interactions (PPIs) and bimolecular fluorescence complementation (BiFC) assay suggested a possible link between VcMYBL1 and VcbHLHL1. Finally, a potential model, in which MYB-bHLH-WD40 complexes play a role in regulating anthocyanin biosynthesis in blueberry fruits, is discussed.

## 2. Materials and Methods

The high quality illumina sequencing reads of green, pink, and blue fruits were submitted to the NCBI short read archive (SRA) database (https://www.ncbi.nlm.nih.gov/sra) (Accession No.: PRJNA546506).

### 2.1. Identification of the MYB-bHLH-WD40 Complex Gene Family Members

To identify the *MYB-bHLH-WD40* complex gene family members, local tBLASTp (https://blast.ncbi.nlm.nih.gov/) (E-value 1 x 10^-5^) searches were performed using amino acid sequences of homologous MYB-bHLH-WD40 complex genes in *Arabidopsis* (*Arabidopsis thaliana*) [19,20], apple (*Malus × domestica*) [31], grape (*Vitis vinifera*) [32,33,34], and strawberry (*Fragaria × ananassa*) [30] (Table S1). The Pfam database (https://pfam.xfam.org/) was used to confirm whether the retrieved genes contained conserved MYB_DNA-binding (PF00249.30, PF13921.5), bHLH-MYC_N (PF14215.5), or WD40 (PF00400.31) domains (Table S2). All the MYB-bHLH-WD40 transcription factors were validated using the PlantTFDB website [36]. The specific genes sequences are shown in Table S3.

### 2.2. Phylogenetic Trees Analysis

**Figure 1:**
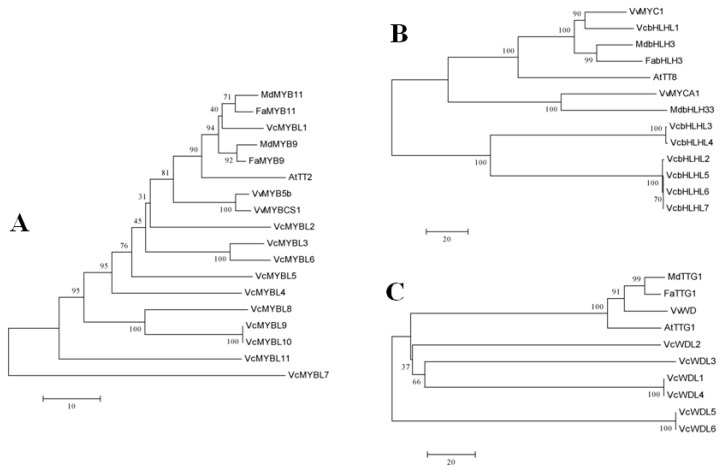
Phylogenetic trees of the *MYB-bHLH-WD40* complex gene family members in blueberry fruits. (**a**) Phylogenetic tree of transcription factor/MYB families, (**b**) phylogenetic tree of transcription factor/basic helix-loop-helix (bHLH) families, (**c**) phylogenetic tree of domain protein/WD-repeat (WD40) families.

All the predicted amino acid sequences of MYB-bHLH-WD40 regulatory complex members were obtained using the NCBI open reading frame (ORF) Finder as in [37]. According to the known MYB-bHLH-WD40 transcription factor genes from *Arabidopsis* (*AtTT2*, *AtTT8*, and *AtTTG1*), apple (*MdMYB11*, *MdMYB9*, *MdbHLH3*, *MdbHLH33*, and *MdTTG1*), grape (*VvMYB5b*, *VvMYBCS1*, *VvMYC1*, *VvMYCA1*, and *VvWD*), and strawberry (*FaMYB11*, *FaMYB9*, *FabHLH3*, and *FaTTG1*) [19,20,30,31,32,33,34], the phylogenetic analysis was executed to determine the relationships (Figure 1). The phylogenetic trees were constructed with the MEGA V5.5 neighbor-joining (NJ) method, using amino acid sequences implementing a *p*-distance model and 1000 bootstrap replicates [38]. Multiple sequence alignments were implemented using the Clustal X software, as described in [39].

### 2.3. Plant Materials and Anthocyanin Analysis

The fruits were randomly sampled at 45 days (green fruits), 65 days (pink fruits), or 85 days (blue fruits) after flowering in the field from 6-year-old healthy blueberry plants. All the samples were snap frozen in liquid nitrogen and stored for subsequent experiments. The anthocyanin of the blueberry fruits was extracted and analyzed using the pH differential method, and the details were as previously reported in [35]. All the experiments were repeated three times.

### 2.4. Differentially Expressed Genes Analysis

To identify the expression patterns of the *MYB-bHLH-WD40* genes, the transcriptome sequencing of the blueberry data during fruit and color development from our previously published study was further explored [35]. The gene expression levels were calculated using the fragments per kilobase per million reads (FPKM) method [40]. *P*-values were adjusted for multiple testing, using the Benjamini–Hochberg false discovery rate (FDR) correction. On the basis of the applied thresholds FDR < 0.01 and log_2_ (foldchange) ≥ 2, the differentially expressed genes (DEGs) analysis was performed by comparing the expression levels.

### 2.5. Subcellular Localization

To determine the subcellular localization of the proteins, the ORF of VcMYBL1, VcbHLHL1, or VcWD40L2 was fused to the N-terminus of the green fluorescent protein (GFP) in the pBI-121 vector, and its expression was driven by the constitutive 35S CaMV promoter. Empty free GFP was used as a control. Protoplast isolation and transformation were performed, as reported previously in Reference [41]. Fluorescence of the GFP in the transformed protoplasts was imagined using confocal laser scanning and it was detected using a laser confocal microscope.

### 2.6. Protein–Protein Interactions (PPIs) Analysis

To determine the interactions of VcMYBL1, VcbHLHL1, and VcWD40L2, the protein–protein interactions (PPIs) analysis was conducted, using the STRING database (version 10.5, http://string-db.org) and grape and *Arabidopsis* as the organisms. The STRING database integrated information from multiple datasets [42]. 

### 2.7. Bimolecular Fluorescence Complementation (BiFC) Analysis

We used the vectors pSPYNE-35S and pSPYCE-35S and the cotransfection vector 35S:P19 to construct a bimolecular fluorescent complementary (BiFC) plasmid vector. For the first time, the VcMYBL1 gene ORF was inserted into the vector pSPYNE-35S, while the VcbHLHL1 gene ORF was inserted into the vector pSPYCE-35S. Both the vectors contained the N- or C-terminus, encoding the yellow fluorescent protein (YFP). Protoplast isolation and transformation were then performed, as previously reported in Reference [41]. Finally, fluorescence of the YFP in the transformed protoplasts was imagined, using confocal laser scanning, and it was detected using a laser confocal microscope.

### 2.8. Quantitative Real-time Reverse Transcription PCR (RT-qPCR)

Quantitative real-time reverse transcription PCR (RT-qPCR) was performed to confirm the DEGs analysis. The blueberry fruits of developmental stages (green, pink, and blue fruits) were sampled. We used the Plant RNA kit (Aidlab-Biotech, Beijing, China) to extract the total messenger RNA (mRNA). The RT-qPCR reactions were performed in a real-time PCR system using SYBR Green (Applied Biosystems, Foster City, CA, USA), according to the manufacturer’s instructions. The *GADPH* house keeping gene was used as a reference, and the RNA relative expression of each gene was calculated using the 2^-ΔΔCT^ method [35,43]. The RT-qPCR reactions were repeated three times. The specific primers are shown in Table S4.

## 3. Results and Discussion

### 3.1. Identification of MYB-bHLH-WD40 Complex Gene Members in Blueberry Fruits

A total of 13 MYB, 8 bHLH, and 8 WD40 unigenes were obtained, using amino acid sequences of homologous MYB-bHLH-WD40 complex genes. A total of two *MYB*, one *bHLH*, and two *WD40* genes of the candidate members were excluded because they did not contain the corresponding conserved domain. The remaining 11 *MYB*, 7 *bHLH*, and 6 *WD40* genes were identified and designated as *VcMYBL1*-*VcMYBL11*, *VcbHLHL1*-*VcbHLHL7*, and *VcWD40L1*-*VcWD40L6*, respectively (Table 1). The subsequently identified *VcMYBL* genes encoded peptides ranging from 129 to 471 amino acids (AAs) with isoelectric point (PI) values varying from 5.22 to 9.97, and molecular weights ranging from 14.91 kD to 52.81 kD, as predicted by the ExPASy server (https://www.expasy.org/). The *VcbHLHL* genes were variable in length, ranging from 468 to 729 AAs with PI values varying from 5.49 to 9.34, and molecular weights ranging from 51.76 to 80.28 kD. The length, PI value, and molecular weight of the identified *VcWD40* genes varied from 254 to 898 AAs, with PI values varying from 4.74 to 8.77, and 28.93 to 100.04 kD, respectively.

### 3.2. Phylogenetic Analyses of the Blueberry MYB-bHLH-WD40 Regulatory Complex Members

The phylogenetic analysis was executed to determine the relationships. As shown in the phylogenetic trees (Figure 1), the highest similarities to the homologous MYB and bHLH TFs were VcMYBL1 and VcbHLHL1. VcMYBL1 had a 56.74% identity to MdMYB11 and 52.7% identity to FaMYB11. VcbHLHL1 had a 92.85% identity to VvMYC1. VcWD40L2 was the most similar to the *WD40* gene compared to other plants.

### 3.3. Expression Patterns of the Blueberry MYB-bHLH-WD40 Genes

**Figure 2:**
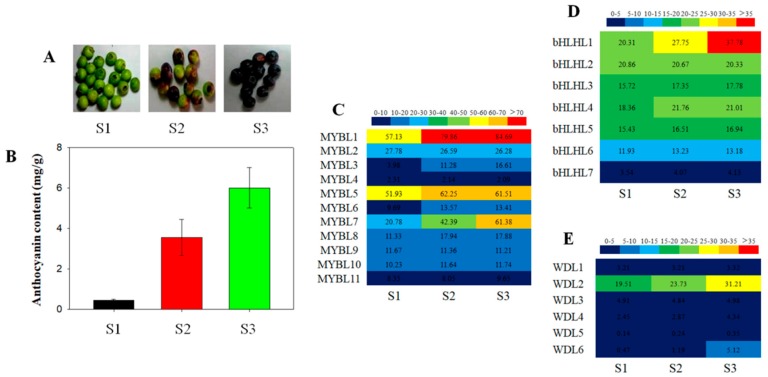
Expression analysis of MYB-bHLH-WD40 complex gene family members in blueberry fruits. (**A**) Blueberry fruit developmental stages. S1, green fruits; S2, pink fruits; S3, blue fruits. (**B**) The content of anthocyanins in blueberry fruits. Error bars are standard errors of the mean from three technical replicates. (**C**) Expression analysis of MYB families. (**D**) Expression analysis of bHLH families. (**E**) Expression analysis of WD40 families. Grids with eight different colors from blue to red show the RNA-seq fragments per kilobase per million reads (FPKM) values.

All the *MYB-bHLH-WD40* genes were expressed in the three blueberry fruit developmental stages: Green (S1), pink (S2), and blue (S3) (Figure 2A). The fruit developmental stages were chosen because of their difference in anthocyanin content. As expected, in the green (S1) developmental stage, anthocyanins were detected at low levels. Based on blueberry fruit growth and ripening, at the fruit’s mature stage (blue) (S3), the level of anthocyanin increases dramatically (Figure 2B).

**Figure 3:**
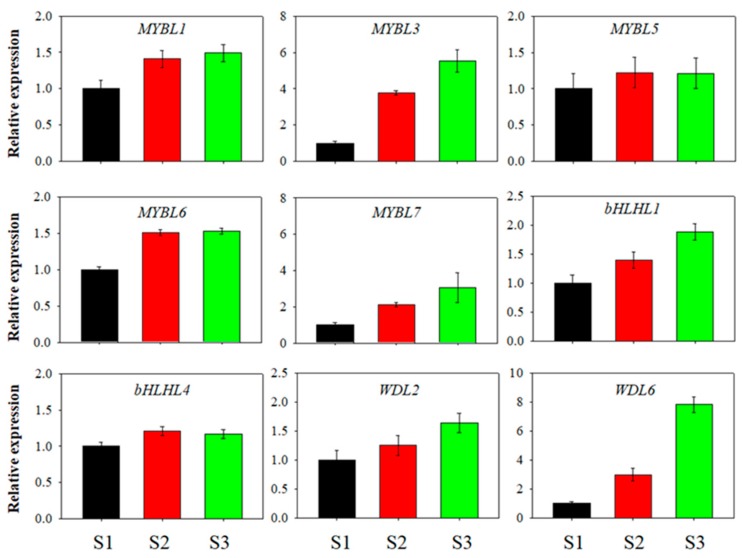
The quantitative real-time reverse transcription PCR (qRT–PCR) validation of RNA-sequencing relative expression estimation. Error bars are standard errors of the mean from three technical replicates.

Among the MYB-bHLH-WD40 genes, some of the *VcMYBL* genes generated the higher level transcripts, especially the *VcMYBL1* genes, while the genes *VcMYBL2*, *VcMYBL4*, etc., remained stable. These results were consistent with previous other plant species findings [44]. We also analyzed the expression patterns of the *VcbHLHL* and *VcWD40L* genes (Figure 2C–E). The qRT–PCR was performed to confirm the results, showing similar trends in the differentially expressed gene (DEG) analysis (Figure 3). The anthocyanin synthesis model of blueberry fruits may be similar to that of *Arabidopsis*, apple, grape, and strawberry. These MYB-bHLH-WD40 complex gene members, *VcMYBL1*, *VcbHLHL1,* and *VcWD40L2*, were identified as being involved in the regulation of the anthocyanin biosynthesis pathway during blueberry fruit ripening and color development [19,20,30,31,32,33,34].

### 3.4. Subcellular Localization of the VcMYBL1, VcbHLHL1, and VcWD40L2 Genes

**Figure 4:**
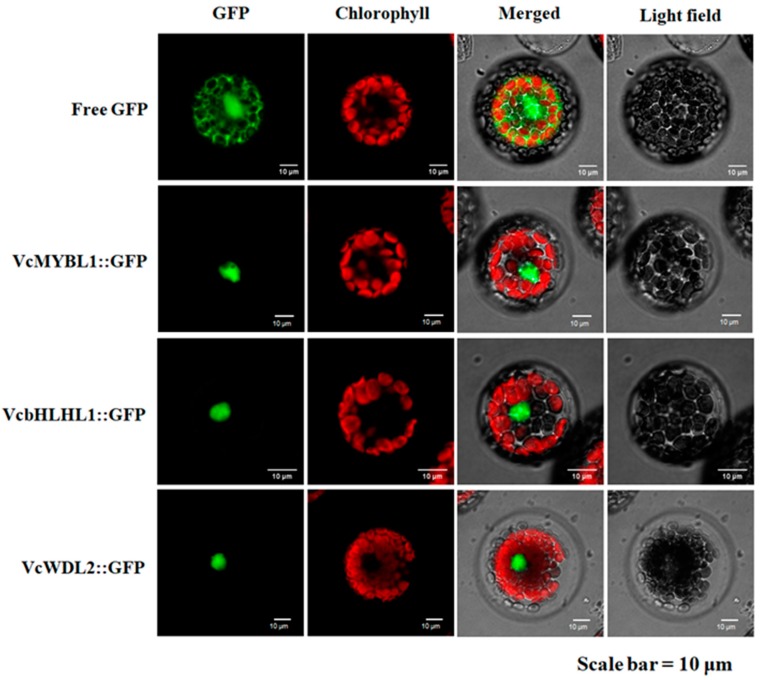
Subcellular localization of free green fluorescent protein (GFP), VcMYBL1::GFP, VcbHLH1::GFP, and VcWDL2::GFP. The chloroplasts have red chlorophyll autofluorescence.

In general, the transcription factors and coactivators must be localized in the nucleus to exert their regulatory effects [37]. In the cells containing the empty free GFP construct, GFP fluorescence was observed in both the cytoplasm and the nucleus, but the GFP fluorescence was detected only in the nuclei of cells containing the VcMYBL1::GFP, VcbHLH1::GFP, and VcWDL2::GFP fusion gene (Figure 4). Therefore, the predicted VcMYBL1::GFP, VcbHLH1::GFP, and VcWDL2::GFP proteins localize to the nucleus, supporting the view that it may be involved in the transcription regulation.

### 3.5. How Expression of MYB-bHLH-WD40 Complexes Can Promote Anthocyanin Biosynthesis in Blueberry Fruits

**Figure 5:**
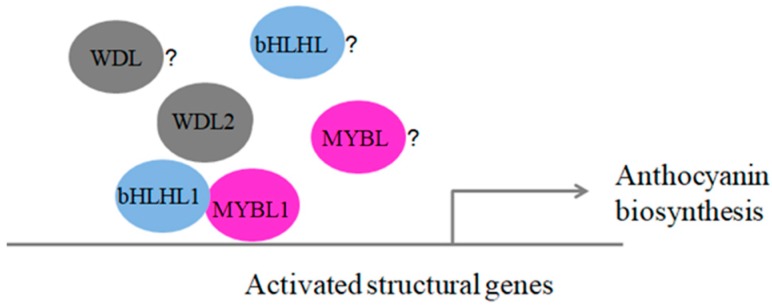
A proposed model for how VcMYBL1, VcbHLHL1, and VcWDL2 expression can promote anthocyanin biosynthesis in blueberry fruits.

How do MYB-bHLH-WD40 complexes regulate the mechanism of anthocyanin biosynthesis pathway genes in blueberry fruits? Through the study of the model plants, *Arabidopsis*, apple, grape, and strawberry, it was shown that the transcription of structural anthocyanin biosynthesis genes was regulated by MYB-bHLH-WD40 complexes [19,20,30,31,32,33,34]. The protein-protein interactions (PPIs) suggested a possible link between VcMYBL1 and VcbHLHL1, but not the VcWD40L2 protein (Figure S1 and Table S5). We used the bimolecular fluorescence complementation (BiFC) assay to validate the hypothesized interactions between the VcMYBL1 and VcbHLHL1 proteins in blueberry fruits (Figure S2). The blueberry VcMYBL1 promotion of anthocyanin biosynthesis was probably achieved by interaction with VcbHLHL1 proteins. The proposed model is depicted in Figure 5. Our future work will involve obtaining experimental evidence confirming this model and verifying whether other MYB-bHLH-WD40 family members are involved in the regulation of anthocyanin synthesis. In summary, to elucidate the role of MYB-bHLH-WD40 complexes in blueberry anthocyanin biosynthesis and molecular regulation mechanisms, it is important to understand the role and function of MYB-bHLH-WD40 complexes in fruit plants, thereby providing an important basis for regulating anthocyanin biosynthesis as well as its breeding.

## 4. Conclusions

Anthocyanins are the main representatives of flavonoids in blueberry fruits. In this study, 11 *MYB*, 7 *bHLH*, and 6 *WD40* genes were identified in blueberry fruits, using the amino acid sequences of homologous MYB-bHLH-WD40 complexes in *Arabidopsis*, apple, grape, and strawberry. The expression patterns of the *MYB-bHLH-WD40* genes were examined using DEGs and qRT–PCR, during blueberry fruit and color development. Consistent with their roles as the transcriptional regulator, VcMYBL1::GFP, VcbHLH1::GFP, and VcWDL2::GFP fusion proteins were observed only in the nucleus. The PPIs and BiFC assay suggested a possible link between VcMYBL1 and VcbHLHL1. A proposed speculation model for how expression of MYB-bHLH-WD40 complexes can promote anthocyanin biosynthesis in blueberry fruits was discussed. These results may provide a foundation to dissect the function of MYB-bHLH-WD40 complexes during blueberry fruit and color development.

## Figures and Tables

**Table 1:** Homologous *MYB-bHLH-WD40* complex gene family members in blueberry fruits.

Name	Deduced Polypeptide				
	Length (aa)	PI	MW (Da)	Pfam	Pfam ID
MYBL1	265	8.57	30057.30	MYB_DNA-binding	PF00249.30, PF13921.5
MYBL2	157	9.71	17780.38	MYB_DNA-binding	PF00249.30, PF13921.5
MYBL3	348	5.37	39436.40	MYB_DNA-binding	PF00249.30, PF13921.5
MYBL4	451	5.58	49663.84	MYB_DNA-binding	PF00249.30, PF13921.5
MYBL5	315	5.22	35681.21	MYB_DNA-binding	PF00249.30, PF13921.5
MYBL6	129	9.00	14908.11	MYB_DNA-binding	PF00249.30, PF13921.5
MYBL7	375	7.12	42961.38	MYB_DNA-binding	PF00249.30, PF13921.5
MYBL8	200	9.26	22052.74	MYB_DNA-binding	PF00249.30, PF13921.5
MYBL9	360	6.26	39085.01	MYB_DNA-binding	PF00249.30, PF13921.5
MYBL10	204	9.97	22644.72	MYB_DNA-binding	PF00249.30, PF13921.5
MYBL11	471	5.82	52808.93	MYB_DNA-binding	PF00249.30, PF13921.5
MYBL12	123	9.36	13594.37		
MYBL13	209	5.33	22759.35		
bHLHL1	729	5.49	80280.20	bHLH-MYC_N	PF14215.5
bHLHL2	589	6.70	64930.88	bHLH-MYC_N	PF14215.5
bHLHL3	491	6.16	54127.08	bHLH-MYC_N	PF14215.5
bHLHL4	491	6.32	54049.06	bHLH-MYC_N	PF14215.5
bHLHL5	589	6.92	65060.05	bHLH-MYC_N	PF14215.5
bHLHL6	468	9.21	51764.32	bHLH-MYC_N	PF14215.5
bHLHL7	498	9.34	55037.05	bHLH-MYC_N	PF14215.5
bHLHL8	371	9.44	40555.56		
WDL1	694	8.77	76244.98	WD40	PF00400.31
WDL2	313	4.74	34289.96	WD40	PF00400.31
WDL3	347	6.41	38427.24	WD40	PF00400.31
WDL4	438	8.73	50248.11	WD40	PF00400.31
WDL5	898	5.67	100036.07	WD40	PF00400.31
WDL6	898	5.67	100036.07	WD40	PF00400.31
WDL7	254	4.80	28932.00		
WDL8	252	4.85	28703.75		

PI: Isoelectric point, MW: Molecular weight, Pfam: Protein family.

## Supplementary Materials

The following are available online at www.mdpi.com/xxx/s1, Figure S1: Protein interaction network of the MYB-bHLH-WD40 proteins, Figure S2: BiFC assays showing the interactions between VcMYBL1 and VcbHLHL1, Table S1: The MYB-bHLH-WD40 gene members in the blueberry RNA-Seq unigenes, Table S2: The MYB-bHLH-WD40 complex gene family members in blueberry fruits, Table S3: *MYB-bHLH-WD40* gene amino acid sequences, Table S4: The primers used for qRT-PCR, Table S5: The potential VcMYBL1, VcbHLHL1, and VcWDL2 protein interactions that were predicted using STRING software.

## References

[1] Galletta G., Ballington B. (1996). Blueberries, cranberries and lingonberries. Fruit Breeding.

[2] Rowland L.J., Alkharouf N., Darwish O., Ogden E.L., Polashock J.J., Bassil N.V., Dorrie M. (2012). Generation and analysis of blueberry transcriptome sequences from leaves, developing fruit, and flower buds from cold acclimation through deacclimation. BMC Plant Biol..

[3] Takashi A., Ayako I., Tomoyuki T., Shozo K., Akihiko S., Atsushi K., Keizo Y. (2009). Dkmyb4 is a myb transcription factor involved in proanthocyanidin biosynthesis in persimmon fruit. Plant Physiol..

[4] Akagi T., Tsujimoto T., Ikegami A., Yonemori K. (2011). Effects of seasonal temperature changes on *dkmyb4* expression involved in proanthocyanidin regulation in two genotypes of persimmon (*diospyros kaki* thunb.) fruit. Planta.

[5] Nijveldt R.J., Van Nood E., Van Hoorn D.E., Boelens P.G., Van Norren K., Van Leeuwen P.A. (2001). Flavonoids: A review of probable mechanisms of action and potential applications. Am. J. Clin. Nutr..

[6] Battino M., Beekwilder J., Denoyesrothan B., Laimer M., Mcdougall G.J., Mezzetti B., Serramajem L., Ngo J., Aranceta J., Solomons N.W. (2010). Bioactive compounds in berries relevant to human health. Nutr. Rev..

[7] Dixon R.A., Xie D.Y., Sharma S.B. (2005). Proanthocyanidins–a final frontier in flavonoid research?. New Phytol..

[8] Cho E., Seddon J.M., Rosner B., Willett W.C., Hankinson S.E. (2004). Prospective study of intake of fruits, vegetables, vitamins, and carotenoids and risk of age-related maculopathy. Arch. Ophthalmol..

[9] Kalt W., Joseph J.A., Shukitt-Hale B. (2007). Blueberries and human health: A review of current reseach. J. Am. Pomol. Soc..

[10] Petroni K., Tonelli C. (2011). Recent advances on the regulation of anthocyanin synthesis in reproductive organs. Plant Sci..

[11] Dubos C., Stracke R., Grotewold E., Weisshaar B., Martin C., Lepiniec L. (2010). MYB transcription factors in *Arabidopsis*. Trends Plant Sci..

[12] Grotewold E. (2005). Plant metabolic diversity: A regulatory perspective. Trends Plant Sci..

[13] Grotewold E. (2006). The genetics and biochemistry of floral pigments. Annu. Rev. Plant Biol..

[14] Koes R., Verweij W., Quattrocchio F. (2013). Flavonoids: A colorful model for the regulation and evolution of biochemical pathways. Trends Plant Sci..

[15] Grotewold E., Sainz M.B., Tagliani L., Hernandez J.M., Bowen B., Chandler V.L. (2000). Identification of the residues in the Myb domain of maize C1 that specify the interaction with the bHLH cofactor R. Proc. Natl. Acad. Sci. USA.

[16] Ben-Simhon Z., Judeinstein S., Nadler-Hassar T., Trainin T., Bar-Ya’Akov I., Borochov-Neori H., Holland D. (2011). A pomegranate (*punica granatum* L.) WD40-repeat gene is a functional homologue of *Arabidopsis TTG1* and is involved in the regulation of anthocyanin biosynthesis during pomegranate fruit development. Planta.

[17] Yongzhen P., Wenger J.P., Katie S., Peel G.J., Jiangqi W., David H., Allen S.N., Yuhong T., Xiaofei C., Million T. (2009). A WD40 repeat protein from *Medicago truncatula* is necessary for tissue-specific anthocyanin and proanthocyanidin biosynthesis but not for trichome development. Plant Physiol..

[18] Pattanaik S., Kong Q., Zaitlin D., Werkman J.R., Xie C.H., Patra B., Yuan L. (2010). Isolation and functional characterization of a floral tissue-specific R2R3 MYB regulator from tobacco. Planta.

[19] Baudry A., Heim M.A., Dubreucq B., Caboche M., Weisshaar B., Lepiniec L. (2010). TT2, TT8, and TTG1 synergistically specify the expression of *BANYULS* and proanthocyanidin biosynthesis in *Arabidopsis thaliana*. Plant J..

[20] Baudry A., Caboche M., Lepiniec L. (2010). TT8 controls its own expression in a feedback regulation involving TTG1 and homologous MYB and bHLH factors, allowing a strong and cell-specific accumulation of flavonoids in *Arabidopsis thaliana*. Plant J..

[21] An J.P., An X.H., Yao J.F., Wang X.N., You C.X., Wang X.F., Hao Y.J. (2018). BTB protein MdBT2 inhibits anthocyanin and proanthocyanidin biosynthesis by triggering MdMYB9 degradation in apple. Tree Physiol..

[22] Cao Y.L., Xie L.F., Ma Y.Y., Ren C.H., Xing M.Y., Fu Z.S., Wu X.Y., Yin X.R., Xu C.J., Li X. (2019). *PpMYB15* and *PpMYBF1* transcription factors are involved in regulating flavonol biosynthesis in peach fruit. J. Agric. Food Chem..

[23] Zhang Q., Hao R.J., Xu Z.D., Yang W.R., Wang J., Cheng T.R., Pan H.T., Zhang Q.X. (2017). Isolation and functional characterization of a R2R3-MYB regulator of *Prunus mume* anthocyanin biosynthetic pathway. Plant Cell. Tiss. Org. Cult..

[24] Wang N., Xu H.F., Jiang S.H., Zhang Z.Y., Lu N.L., Qiu H.R., Qu C.Z., Wang Y.C., Wu S.J., Chen X.S. (2017). MYB12 and MYB22 play essential roles in proanthocyanidin and flavonol synthesis in red-fleshed apple (*Malus sieversii* f. niedzwetzkyana). Plant J..

[25] Liu C.Y., Long J.M., Zhu K.J., Liu L.L., Yang W., Zhang H.Y., Li L., Xu Q., Deng X.X. (2016). Characterization of a Citrus R2R3-MYB transcription factor that regulates the flavonol and hydroxycinnamic acid biosynthesis. Sci. Rep..

[26] Grotewold E., Drummond B.J., Bowen B., Peterson T. (1994). The *myb* -homologous *P* gene controls phlobaphene pigmentation in maize floral organs by directly activating a flavonoid biosynthetic gene subset. Cell.

[27] Petroni K., Cominelli E., Consonni G., Gusmaroli G., Gavazzi G., Tonelli C. (2000). The developmental expression of the maize regulatory gene *Hopi* determines germination-dependent anthocyanin accumulation. Genetics.

[28] Falcone Ferreyra M.L., Rius S., Emiliani J., Pourcel L., Feller A., Morohashi K., Casati P., Grotewold E. (2010). Cloning and characterization of a UV-B-inducible maize flavonol synthase. Plant J..

[29] Feller A., Machemer K., Braun E.L., Grotewold E. (2011). Evolutionary and comparative analysis of MYB and bHLH plant transcription factors. Plant J..

[30] Schaart J.G., Dubos C., Romero D.L.I., Van Houwelingen A.M., Vos R.C., Jonker H.H., Xu W., Routaboul J.M., Lepiniec L., Bovy A.G. (2013). Identification and characterization of MYB-bHLH-WD40 regulatory complexes controlling proanthocyanidin biosynthesis in strawberry (*Fragaria × ananassa*) fruits. New Phytol..

[31] An X.H., Tian Y., Chen K.Q., Wang X.F., Hao Y.J. (2012). The apple WD40 protein MdTTG1 interacts with bHLH but not MYB proteins to regulate anthocyanin accumulation. J. Plant Physiol..

[32] Deluc L., Barrieu F., Marchive C., Lauvergeat V., Decendit A., Richard T., Carde J.P., Mérillon J.M., Hamdi S. (2006). Characterization of a grapevine R2R3-MYB transcription factor that regulates the phenylpropanoid pathway. Plant Physiol..

[33] Deluc L., Bogs J., Walker A.R., Ferrier T., Decendit A., Merillon J.M., Robinson S.P., Barrieu F. (2008). The transcription factor VvMYB5b contributes to the regulation of anthocyanin and proanthocyanidin biosynthesis in developing grape berries. Plant Physiol..

[34] Terrier N., Torregrosa L., Ageorges A., Vialet S., Verriès C., Cheynier V., Romieu C. (2009). Ectopic expression of VvMybPA2 promotes proanthocyanidin biosynthesis in grapevine and suggests additional targets in the pathway. Plant Physiol..

[35] Li L., Zhang H., Liu Z., Cui X., Zhang T., Li Y., Zhang L. (2016). Comparative transcriptome sequencing and *de novo* analysis of *Vaccinium corymbosum* during fruit and color development. BMC Plant Biol..

[36] Jin J., Zhang H., Kong L., Gao G., Luo J. (2014). PlantTFDB 3.0: a portal for the functional and evolutionary study of plant transcription factors. Nucleic Acids Res..

[37] Liu L., White M.J., MacRae T.H. (1999). Transcription factors and their genes in higher plants functional domains, evolution and regulation. Eur. J. Biochem..

[38] Tamura K., Peterson D., Peterson N., Stecher G., Nei M., Kumar S. (2011). MEGA5: Molecular evolutionary genetics analysis using maximum likelihood, evolutionary distance, and maximum parsimony methods. Mol. Biol. Evol..

[39] Thompson J.D., Gibson T.J., Plewniak F., Jeanmougin F., Higgins D.G. (1997). The CLUSTAL_X windows interface: flexible strategies for multiple sequence alignment aided by quality analysis tools. Nucleic Acids Res..

[40] Trapnell C., Williams B.A., Pertea G., Mortazavi A., Kwan G., Van Baren M.J., Salzberg S.L., Wold B.J., Pachter L. (2010). Transcript assembly and quantification by RNA-Seq reveals unannotated transcripts and isoform switching during cell differentiation. Nat. Biotechnol..

[41] Yoo S., Cho Y., Sheen J. (2007). *Arabidopsis* mesophyll protoplasts: A versatile cell system for transient gene expression analysis. Nat. Protoc..

[42] Szklarczyk D., Franceschini A., Kuhn M., Simonovic M., Roth A., Minguez P., Doerks T., Stark M., Muller J., Bork P. (2011). The STRING database in 2011: functional interaction networks of proteins, globally integrated and scored. Nucleic Acids Res..

[43] Livak K.J., Schmittgen T.D. (2001). Analysis of relative gene expression data using real-time quantitative PCR and the 2 ^−ΔΔCT^ method. Methods.

[44] Shi L., Chen X., Chen W., Zheng Y., Yang Z. (2018). Comparative transcriptomic analysis of white and red Chinese bayberry (*Myrica rubra*) fruits reveals flavonoid biosynthesis regulation. Sci. Hortic..

