# Supplementary material for: Identification and Characterization of MYB-bHLH-WD40 Regulatory Complex Members Controlling Anthocyanidin Biosynthesis in Blueberry Fruits Development
Source: Genes (Basel). 2019 Jun 28;10(7):496. doi: 10.3390/genes10070496 (PMC6678982; doi:10.3390/genes10070496)
Supplement: Supplementary file 1 [file genes-10-00496-s001.pdf]

Supplementary information

Title: Identification and characterization of MYB-bHLH-WD40 regulatory complex members controlling anthocyanidin biosynthesis in blueberry fruits development

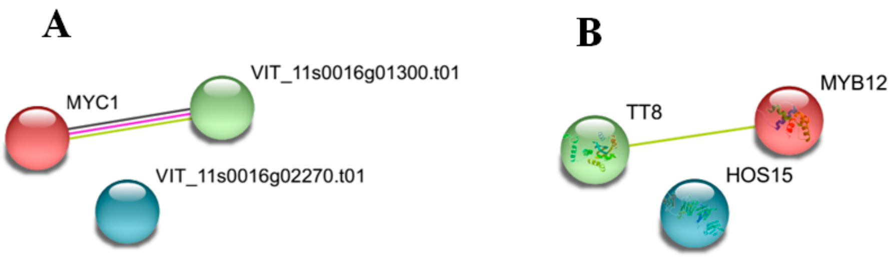

Figure S1: Protein interaction network of MYB-bHLH-WD40 proteins. The protein-protein interactions (PPIs) analysis were conducted using STRING (version 10.5, http://string-db.org) and grape (A) and *Arabidopsis* (B) as the organism. In the resulting proteins network, proteins are represented by nodes which are linked by lines. Various line colors denote different kinds of associations: red line represents fusion association evidence; green line represents neighborhood association; light blue line indicates database evidence; black line represents co-expression evidence; purple line denotes experimental evidence of proteins association; yellow line represents text mining evidence and blue line indicates co-occurrence

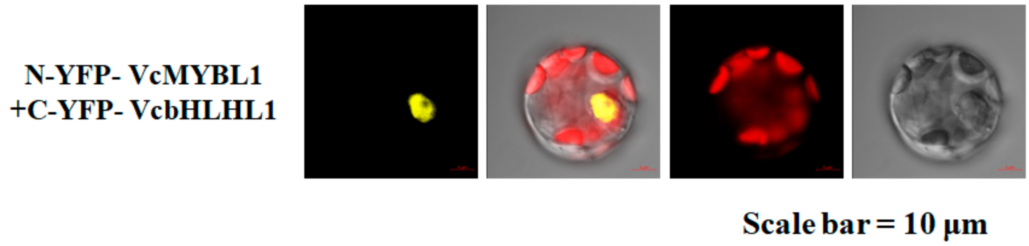

Figure S2: BiFC assays showing the interactions between VcMYBL1 and VcbHLHL1. The chloroplasts have red chlorophyll autofluorescence.

Table S1: The MYB-bHLH-WD40 gene members in the blueberry RNA-Seq unigenes

Unigene	Bit score	E-value	Designated
c37651	238	5.00E-75	MYBL1
c104049	148	1E-40	MYBL2
c27453	146	4E-40	MYBL3
c101736	139	1E-36	MYBL4
c115818	137	1E-36	MYBL5
c30681	129	2.00E-33	MYBL6
c116957	100	4.00E-23	MYBL7
c115306	97.4	3.00E-22	MYBL8
c12778	95.9	7.00E-22	MYBL9
c12840	92.4	2.00E-20	MYBL10
c16862	71.6	2E-13	MYBL11
c65826	149	4.00E-41	MYBL12
c717177	68.6	3E-12	MYBL13
c96530	228	3.00E-64	bHLHL1
c99067	78.2	3E-14	bHLHL2
c101814	77.4	3E-14	bHLHL3
c26425	76.6	6E-14	bHLHL4
c100600	74.7	3E-13	bHLHL5
c106224	61.6	3E-09	bHLHL6
c109989	61.6	4E-09	bHLHL7
c94182	226	8E-63	bHLHL8
c12480	54.3	2.00E-07	WDL1
c109016	53.1	3.00E-07	WDL2
c14545	51.6	1.00E-06	WDL3
c1740	49.7	6.00E-06	WDL4
c30377	49.3	6.00E-06	WDL5
c57901	49.3	6.00E-06	WDL6
c96084	294	1.00E-91	WDL7
c106660	287	3.00E-89	WDL8

Table S2: The MYB-bHLH-WD40 complex gene family members in blueberry

Name	Start	End	Pfam	Pfam id	Bit score	E-value
MYBL1	14	61	PF00249.30	Myb_DNA-binding	54.73	7.5E-15
MYBL1	67	112	PF00249.30	Myb_DNA-binding	55.76	3.6E-15
MYBL1	17	76	PF13921.5	Myb_DNA-binding	48.11	9.4E-13
MYBL1	71	124	PF13921.5	Myb_DNA-binding	30.88	0.00000023
MYBL2	14	61	PF00249.30	Myb_DNA-binding	54.79	7.2E-15
MYBL2	67	112	PF00249.30	Myb_DNA-binding	51.91	5.7E-14
MYBL2	17	76	PF13921.5	Myb_DNA-binding	41.02	1.5E-10
MYBL2	70	118	PF13921.5	Myb_DNA-binding	28.82	0.00000099
MYBL3	14	61	PF00249.30	Myb_DNA-binding	54.54	8.6E-15
MYBL3	67	112	PF00249.30	Myb_DNA-binding	52.4	4E-14
MYBL3	17	75	PF13921.5	Myb_DNA-binding	36.1	5.3E-09
MYBL3	71	118	PF13921.5	Myb_DNA-binding	25.94	0.0000079
MYBL4	23	70	PF00249.30	Myb_DNA-binding	49.51	3.2E-13
MYBL4	76	119	PF00249.30	Myb_DNA-binding	53.11	2.4E-14
MYBL4	26	85	PF13921.5	Myb_DNA-binding	45.03	8.6E-12
MYBL4	79	122	PF13921.5	Myb_DNA-binding	24.19	0.000028
MYBL5	35	82	PF00249.30	Myb_DNA-binding	60.77	9.8E-17
MYBL5	88	131	PF00249.30	Myb_DNA-binding	54.24	1.1E-14
MYBL5	38	96	PF13921.5	Myb_DNA-binding	49.69	3E-13
MYBL5	94	134	PF13921.5	Myb_DNA-binding	25.34	0.000012
MYBL6	14	61	PF00249.30	Myb_DNA-binding	53.09	2.4E-14
MYBL6	67	105	PF00249.30	Myb_DNA-binding	43.56	2.3E-11
MYBL6	17	76	PF13921.5	Myb_DNA-binding	43.01	3.7E-11
MYBL7	181	227	PF00249.30	Myb_DNA-binding	63.81	1.1E-17
MYBL7	233	275	PF00249.30	Myb_DNA-binding	59.95	1.8E-16
MYBL7	184	241	PF13921.5	Myb_DNA-binding	65.09	4.7E-18
MYBL7	236	276	PF13921.5	Myb_DNA-binding	37.09	2.6E-09
MYBL8	65	110	PF00249.30	Myb_DNA-binding	57.04	1.4E-15
MYBL8	119	161	PF00249.30	Myb_DNA-binding	58.48	5E-16
MYBL8	68	126	PF13921.5	Myb_DNA-binding	55.07	6.3E-15
MYBL8	120	169	PF13921.5	Myb_DNA-binding	34.27	0.00000002
MYBL9	12	57	PF00249.30	Myb_DNA-binding	70.48	9E-20
MYBL9	66	108	PF00249.30	Myb_DNA-binding	53.82	1.4E-14
MYBL9	15	72	PF13921.5	Myb_DNA-binding	61.45	6.5E-17
MYBL9	67	114	PF13921.5	Myb_DNA-binding	36.1	5.3E-09
MYBL10	12	57	PF00249.30	Myb_DNA-binding	71.72	3.7E-20
MYBL10	66	108	PF00249.30	Myb_DNA-binding	55.04	6E-15
MYBL10	15	72	PF13921.5	Myb_DNA-binding	62.14	3.9E-17
MYBL10	67	115	PF13921.5	Myb_DNA-binding	37.47	0.000000002
MYBL11	13	57	PF00249.30	Myb_DNA-binding	46.96	2E-12
MYBL11	63	106	PF00249.30	Myb_DNA-binding	45.85	4.4E-12
MYBL11	14	73	PF13921.5	Myb_DNA-binding	50.4	1.8E-13

MYBL11	66	117	PF13921.5	Myb_DNA-binding	29.86	0.00000047
bHLHL1	9	191	PF14215.5	bHLH-MYC_N	195.01	1.1E-57
bHLHL2	52	240	PF14215.5	bHLH-MYC_N	187.92	1.6E-55
bHLHL3	48	229	PF14215.5	bHLH-MYC_N	160.7	3.6E-47
bHLHL4	48	229	PF14215.5	bHLH-MYC_N	160.59	3.9E-47
bHLHL5	52	240	PF14215.5	bHLH-MYC_N	186.78	3.6E-55
bHLHL6	1	119	PF14215.5	bHLH-MYC_N	123.96	7E-36
bHLHL7	24	149	PF14215.5	bHLH-MYC_N	130.49	6.9E-38
WDL1	150	183	PF00400.31	WD40	18.34	0.0028
WDL1	189	226	PF00400.31	WD40	20.79	0.00048
WDL1	231	268	PF00400.31	WD40	30.7	3.50E-07
WDL1	273	310	PF00400.31	WD40	33.13	6.00E-08
WDL1	316	352	PF00400.31	WD40	20.24	0.00071
WDL1	364	395	PF00400.31	WD40	21.06	0.00039
WDL2	105	139	PF00400.31	WD40	20.37	0.00065
WDL2	195	230	PF00400.31	WD40	15.62	0.02
WDL3	56	86	PF00400.31	WD40	25.59	1.40E-05
WDL3	90	127	PF00400.31	WD40	21.48	0.00029
WDL3	173	210	PF00400.31	WD40	38.54	1.20E-09
WDL3	217	261	PF00400.31	WD40	20.62	0.00054
WDL3	267	303	PF00400.31	WD40	39.58	5.50E-10
WDL3	307	344	PF00400.31	WD40	12.22	0.24
WDL4	221	254	PF00400.31	WD40	19.14	0.0016
WDL4	260	297	PF00400.31	WD40	21.59	0.00027
WDL4	302	339	PF00400.31	WD40	31.5	2.00E-07
WDL4	344	381	PF00400.31	WD40	33.93	3.40E-08
WDL4	387	423	PF00400.31	WD40	21.04	0.0004
WDL5	588	615	PF00400.31	WD40	12.13	0.26
WDL5	676	709	PF00400.31	WD40	20.73	0.0005
WDL5	758	793	PF00400.31	WD40	19.33	0.0014
WDL6	588	615	PF00400.31	WD40	12.13	0.26
WDL6	676	709	PF00400.31	WD40	20.73	0.0005
WDL6	758	793	PF00400.31	WD40	19.33	0.0014

Table S3: MYB-bHLH-WD40 genes amino acid sequences

Gene	sequences
MYBL1	MGRSPCCAKEGLNRGSWTASEDRILTDYVKLHGEGDWRNLPKKAGLKRCGKSCRLRWLNYLRPDIKRGNI<br>SPDEEELIIRLHKLLGNRWSLIAGRLPGRTDNEIKNYWNTNLCKKVQDHHCSDKSVKNKRKKPKMEPNIES<br>KVVRTKAMRCTKVFISPQLQKIESIDHNKGPPVPESISCNIGLSQFAPKDDNPTLDDLMFDVNMEDVFMS<br>GILNSDFDGTCFFNYGGDKFDDLSASVPDGTLFCSKDMLEDWTLNNCLQSNGVG
MYBL2	MGRPPCCDKGGVKKGPWTPEEDIVLVTYIQDHGPGNWRAVPTNTGLLRCSKSCRLRWTNYLRPGIKRGN<br>FSDHEEKMIIHLQALLGNRWAAIASYLPQRTDNDIKNYWNTHLKKKLKKLQGQDGHFTDGFSPILCPPPRV<br>SGREGSKQTSTWQASLV
MYBL3	MGRSSEETKPKLRKGLWSPEEDEKLFNYISRFGVGCWSAVPKLAGLERCGKSCRLRWMNYLRPDLKRGM<br>FSLQEEDLIITLHEALGNRWAQIAAQLPGRTDNEIKNFWNSYLKKKLMKQGTDPITHKAISEIEEIEWKNPA<br>EKSPLGLPRLKVDCAVVSPIWPCPERISKLRKNEVVPTTVSMEQEFHVTNTNYYEGGGRDASIEKLGTKPN<br>FDPLVLYEFQGGPDPIGYNSNLLTNYDHQTHFEDNPNFAFAPGPALTDLSDNSSLRISSFSFNEMKESSSNSS<br>NASIVENNGTFSCGEELVFGFQCEEMKPSMWPEGQIGHNSESFSSYPLMSVSESMDFSSKYYM
MYBL4	MVASSGSGSRDEGGGGSRKAVKKGPWTTAEDAMLMEYVRKNGEGNWNAVQRNCGLMRCGKSCRLW<br>ANHLRPNLKKGSFSAEEERLIIELHAHLGNKWARMASQLPGRTDNEIKNYWNTRVKRHQRAGLPIYPDDI<br>QQQQYDQYSQLHQQRSYHKSKFNPSLPLLDPMTNFPTIPNPTQPPCPSSNFLSNPSHQFTFFRNTERSSR<br>DFSLPQSSPFSPSSLFNQSLIKTEYLPPYPVHQFRHDDFEINPFAIFPPCNETELPSIQSPAVAMPPTPTTSGT<br>TCGGDYTVIGTTGDAPDQTAANSGLLDALLEESNALVNAENLRNEVSFANSSLQPGFIEDDGGDFFNDSSS<br>ANSSVGMKKKEPILLEEMNHSEDDFSSLFNNFPLTDPVPPWHNASADSSNGKPSNVKEYGDSAINNSPEA<br>SHTLGETALATGSPSWSLGSGSWNNMPGIC
MYBL5	MEVYESRGNCNPNDYSINLSDQEDGQEQDQMEMRRGPWTVEEDLMLMNYVANHGEGRWNSLARCA<br>GLKRTGKSCRLRWLNYLRPDVRRGNISLEEQLLILELHSRWGNRWSKIAQHLPGRTDNEIKNYWRTRVQK<br>HAKQLKCDVNSKQFKDTMRYLWMPRLVERIQAASAASGGSTTTTTTTTAAAGQVIPPPGVFTGDFGGAQ<br>VNKSYTPENSSTGASSDSFGAQVSPASDLTDYYSNYPVNSNYSNNQNYYQVNQGNYSDSLTSPSGYFNPG<br>LDFQAVEQNSQQQLFDAAGDISDNLWDVEDIWFLQQQLNSM
MYBL6	MGRQSCYVKPKLRKGLWSPEEDEKLFNHITRFGVGCWSSVPKLAGLQRCGKSCRLRWINYLRPDLKRGM<br>FSQQEEDLILSLHESLGNRWAQIAAQLPGRTDNEIKKLLELFFEEETNETGGLIQTPTSR
MYBL7	MEFDSTNFKENPHFHQSFSQNRSKPQMENGLLMGSSPSKGFYQSFTNFDQVSNAENSFSNFPFGNLTPF<br>HDPFDPFIASSSTPPSDQCGNFFPTSESKNPSGLNGVKMGLFMSYSPRILLRSLNTEENSQKLQQMQSNA<br>SVEVPCLNVEDMYQAKANLEKKKKDLTKESEKAFQNSPILVKGQWSPEEDRLLVQLVNQYGERKWSLIAE<br>MLDGRVGKQCRERWHNHLRPGIKKDRWTEEEEMILIEAHKKMGNKWAAIARELPGRSENTVKNHWNA<br>AKRRRFNTKRKCKYNSLLRQYIMSLSSPDQSSEMLHFKDQMEPTNCESDHNSKAVDGSSELGQSIFGYQV<br>FDKMAELGREIGLGVGEMDLLEMVRQGII
MYBL8	MSSDAEMDDAPVVHGVAPALDDGPSTATSAGDVEGQNDEVFDAAAVGEGRGGGRRAGKKYGDRVKG<br>PWSPEEDAVLSRLVSNFGARNWSLIARGIEGRSGKSCRLRWCNQLDPNVMRIPFTDEEDRLIVKAHAIHG<br>NKWASIARLLPGRTDNAIKNHWNSTLRRRSMGRSSSIWNPVLWQKTLASTNPKHHLRTLYQMEI
MYBL9	MASTKKDVDRIKGPWSPEEDEQLQNLVQKHGPRNWSLISKSIPGRSGKSCRLRWCNQLSPQVEHRAFTP<br>EEDDTIIRAHARFGNKWATIARLLSGRTDNAIKNHWNSTLKRKCSSMMGNDGDEFSNTPPLKRSVSDGSA<br>IPVSGLYLSPNSPSGSDVSDSSVPPPPPPPPVFSSSSSSSSHVFRPLARTGGVLPPQVETTTPSSMNTSLSLSL<br>PGVDSSDPPSQVTESIPIQLLPMLPVYPAAATPEQVTVNQRNQEAPEVRVPVMAFPVNLRESDVREPAST<br>PVAIEVEREEKAFVPFSAEFLAVMQEMIRKEVWNYMAEVEQQGGGGSGGGGGFCMQQATVDGFRNAI<br>VKRMGIGRVE

MASTKKDVDRIKGPWSPEEDEQLQNLVQKHGPRNWSLISKSIPGRSGKSCRLRWCNQLSPQVEHRAFTP
MYBL10 EEDDTIIRAHARFGNKWATIARLLSGRTDNAIKNHWNSTLKRKCSSMMGNDGDEFSNTPPLKRSVSDGSA
IPVSGLYLSPNSPSGSDVSDSSVPPPPPPPPVSPPPPLLPLTCSVLWRGPGAFCRLRWKPQRPRR

MENMKKKERHIVSWSQEEDDILREQIRINGTENWAIIASNFKDKTTRQCRRRWYTYLNSDFKKGGWSPE
EDVLLCEAQKIFGNRWTEIAKVVSGRTDNAVKNRFSTLCKKRAKHEALAKENTTSYINSNNKRVILEDGFKP
AGIPDTAISLKKMRRTHLSDLTEKCNNGERLTGECGITSQQLRPPFAVLVQNFHNVGSLPSQHHVSNSTENP
MYBL11 TDGTASNDKIQGTFLKKDDPRILALMQQAELLSSLAVKVNTENTDQSLENAWKVLQEFLDQNKGSDMLSI
KISDMDFQFENFKDLVEDLRSGSDCNQPSWRQPDLFEESQGSSDFSTGSTVIPQTAGDKTQHSQGKLCAQ
HQDTLFESQSIPIGDQNSIGDCQNVFSGATSTQDMLPSCDDVNDHDGIMVAFSNTEFGSPLQVTPIFRSLS
SAVPSPKFSESERQFLMKALRVESPSCNPSNNASQPPPCRRALLQSL

MAAPPSTRLQNMLQTAVQSVQWTYSLFWQLCPQQGILVWGDGYYNGAIKTRKTVQPVEVSAEEASLQR
SQQLRELYDSLSAGESNQQTRRPCASLSPEDLTESEWFYLMCVSFSFPPGVGLPGKAYAKRQHVWLTGAN
EVDSKVFSRAILAKSARIQTVVCIPLLDGVVELGTTERVQEDIGLVQQVKSFFIDHHPPQPPKPALSEHSTSN
PATSSDHPRFHSPTVPNVHAPVNPPVNPNQIDEEEEEEEEDEEDEEEEEEEGESESEADTGRNSHLSGARN
PRVVNVAQAAAEPSELMQLEMSEDIRLGSPDDGSNNLDSEFQLLAASQGGNAVDHQRRADSYRAESTR
bHLHL1 RWPLLQDPMSSNLQPPPSGGPALEELTQEDTHYSQTVSTILQHQSTRWSDSSTSSAAAAPGYLMYSSQSS
FSKWNLHPSDHHHYAASAADGTSQWLLKYILFSVPFLHTKYHDNNSNSPKSASAAANDSASRFRKTTPQD
ELSANHVLAERRRREKLNERFIILRSLVPFVTKMDKASILGDTIEYVKQLRKKIQDLESRTRQMESDQRSRSS
GDLQRTSSLSLKEQRSGVTTVVNTDRANRVGGGPTGSDKRKLRIVEGGSGGAKGKAVDSSPVEVPPSPPP
PPPPPQPVAGVGLQVQVSIIESDALVELQCPHREGLLLDVMVVLRDHRVEVTAVQSSLTNGVFVAELRAKV
KDNGSGKKPSIVEVKRAIHQIIPSY

MKVEVCMGGVGWSEEDKAMGAAVLGTKGFDYLISSSVSAECSLMSVGSDENLQNKLSDLVDQPDASNF
SWNYAFFWQISRSKAGDLVLGWGDGSCREPREGEESELTQIAHSRLEEVTQQRMRKRVLQKLHTLFGGS
DEDSYAFGLDKVTDTEMLFLASMYFSFPRGEGGPGKCFASGKHVWISNSLKASSDYCVRSFLAKSAGIQTIV
LIPTDVGVVEFGSMRSIPENFELVKSIRSSFSSFSSLAKAKPTKDVNAHFFNLGNGERPDGIPKIFGQDLCSG
bHLHL2 RSQFREKLAVRKAGEKPWEAYSNGSRSPFPNARHGLNGSSWTQFQSVKQVATAGIYSPHTPSNNLPEVIN
GVRDEFRLNNYQSQKAAATMQIDFTGGATSRPPVTSRPASVESEHSDVEASGKDELAGAVDEKRPRKRGR
KPANGREEPLNHVEAERQRREKLNQRFYALRAVVPNISKMDKASLLGDAIAYITELQKKLKDMESDRENLG
ANPISENQTRVPHIEIQATGHDEVVVRVSCPSDTHPVSRVIQAFKEAQITVLESKLAVGNETIFHTFVIKSQG
SEQITKERLVAAFSHESNSMQPL

MGEKFWLNEVDKGMVESVLGREAFEFLVASASGNVLSEFASSAGDLGLQKGLSKIVEGSKWNYAIFWQVS
KSKSGNSALIWGDGYCREAKGGEVGTGNGLQDRKFEGGDRKRRVLQKLHACFGGVEEDNCALNLDLVSD
VEMFYLTSMYYSFPFDKPSTPSQSFNTGRVIWASDTKSCSENYQSRSYLANMARFETVVFVPLKSGVVEIG
bHLHL3 SVKPIPEEQNLVQKVKTTFGGPLPSQAKLLPKIFGHELSLGGAKSRPMTISFSPKVEDDSGFASESFELQGVG
SNQVYGNSSNGCRSDEGEAKLFPHMNLGGLEQGQEDLLIQSDERKPRKRGRKPANGREEPLNHVEAERQ
RREKLNQRFYALRAVVPNISKMDKASLLGDAISYITDLQGKIRILEAEKDMVNYKQKQCTVPEIDFQARHDD
AVVRVSCPLDSHPVSTVVKALREQQCIAQESNVSTTEEGEIVHTFSIRAQGGAAEHLKEKLVAALSI

MGEKFWLNEVDKGMVESVLGREAFEFLVASASGNVLSEFASSAGDLGLQKGLSKIVEGSKWNYAIFWQVS
KSKSGNSALIWGDGYCREAKGGEVGTGNGLQDRKLEGGDRKRRVLQKLHACFGGVEEANCALNLDLVSD
VEMFYLTSMYYSFPFDKPSTPSQSFNTGRVIWASDTKSCSENYQSRSYLANMARFETVVFVPLKSGVVEIG
bHLHL4 SVKPIPEEQNLVQKVKTTFGGPLPSQAKLLPKIFGHELSLGGAKSRPMTISFSPKVEDDSGFASESFELQGVG
SNQVYGNSSNGCRSDEGEAKLFPHMNLGGLEQGQEDLLIQSDERKPRKRGRKPANGREEPLNHVEAERQ
RREKLNQRFYALRAVVPNISKMDKASLLGDAISYITDLQGKIRILEAEKDMVNYKQKQCTVPEIDFQARHDD
AVVRVSCPLDSHPVSTVVKALREQQCIAQESNVSTTEEGEIVHTFSIRAQGGAAEHLKEKLVAALSI

bHLHL5

MKVEVCMGGVGWSEEDKAMGAAVLGTKGFDYLISSSVSAECSLMSVGSDENLQNKLSDLVDQPDTSNF
SWNYAFFWQISRSKAGDLVLGWGDGSCREPREGEESELTQIAHSRLEEVTQQRMRKRVLQKLHTLFGGS
DEDSYAFGLDKVTDTEMLFLASMYFSFPRGEGGPGKCFASRKHVWISNSLKASSDYCVRSFLAKSAGIQTIV
LIPTDVGVVEFGSMRSIPENFELVKSIRSSFSSFSSLAKAKPTKDVNAHFFNLGNGERPDGIPKIFGQDLCSG
RSQFREKLAVRKAGEKPWEAYSNGSRSPFPNARHGLNGSSWTQFQSVKQVATAGIYSPHTPSNNLPEVIN
GVRDEFRLNNYQSQKAAATMQIDFTGGATSRPPVTSRPASVESEHSDVEASGKDELAGAVDEKRPRKRGR
KPANGREEPLNHVEAERQRREKLNQRFYALRAVVPNISKMDKASLLGDAIAYITELQKKLKDMESDRENLG
ANPISENQTRVPHIEIQATGHDEVVVRVSCPSDTHPVSRVIQAFKEAQITVLESKLAVGNETIFHTFVIKSQG
SEQITKERLVAAFSHESNSMQPL

bHLHL6

MRKRVLQKLHTLFGGSDEDSYAFGLDKVTDTEMLFLASMYFSFPRGEGGPGKCFASRKHVWISNSLKASS
DYCVRSFLAKSAGIQTIVLIPTDVGVVEFGSMRSIPENFELVKSIRSSFSSFSSLAKAKPTKDVNAHFFNLGNG
ERPDGIPKIFGQDLCSGRSQFREKLAVRKAGEKPWEAYSNGSRSPFPNARHGLNGSSWTQFQSVKQVATA
GIYSPHTPSNNLPEVINGVRDEFRLNNYQSQKAAATMQIDFTGGATSRPPVTSRPASVESEHSDVEASGKD
ELAGAVDEKRPRKRGRKPANGREEPLNHVEAERQRREKLNQRFYALRAVVPNISKMDKASLLGDAIAYITE
LQKKLKDMESDRENLGANPISENQTRVPHIEIQATGHDEVVVRVSCPSDTHPVSRVIQAFKEAQITVLESKL
AVGNETIFHTFVIKSQGSEQITKERLVAAFSHESNSMQSL

bHLHL7

MGLVGSQGKGRSLNLPQIAHSRLEEVTQQRMRKRVLQKLHTLFGGSDEDSYAFGLDKVTDTEMLFLASM
YFSFPRGEGGPGKCFASRKHVWISNSLKASSDYCVRSFLAKSAGIQTIVLIPTDVGVVEFGSMRSIPENFELV
KSIRSSFSSFSSLAKAKPTKDVNAHFFNLGNGERPDGIPKIFGQDLCSGRSQFREKLAVRKAGEKPWEAYSN
GSRSPFPNARHGLNGSSWTQFQSVKQVATAGIYSPHTPSNNLPEVINGVRDEFRLNNYQSQKAAATMQI
DFTGGATSRPPVTSRPASVESEHSDVEASGKDELAGAVDEKRPRKRGRKPANGREEPLNHVEAERQRREK
LNQRFYALRAVVPNISKMDKASLLGDAIAYITELQKKLKDMESDRENLGANPISENQTRVPHIEIQATGHDE
VVVRVSCPSDTHPVSRVIQAFKEAQITVLESKLAVGNETIFHTFVIKSQGSEQITKERLVAAFSHESNSMQSL

WDL1

MMRQSSALSSNMGGQPDYHHQPLAQPPHPPYDAQGDSFAAKRIRKIGQRRAVDYTSTVVRYLQIRMFQ
RDSRDRTVLQPTPAAAIDMLPAVAYSDNPSTSFASKFVHTSLNKNRCSINRVLWTPSGRRLITGSQSGEFTL
WNGQSFNFEMILQAHDQAVRSMVWSYNENWMVTGDDGGSIKYWQNNMNNVKAYKSAHKESVRDL
SFCRTDLKFCSCSDDTTVKVWDFARCQEERSLSGHGWDVKTVDWHPTKSLLASGGKDNLVKLWDAKSG
RELSSFHGHKNTVLCVKWNQNGNWVLTASKDQIIKLYDIRAMKELESFRGHQKDVTALAWHPFHEEYFVS
GSLDGSIFHWLVGHDTPQVEIPNAHDNSVWDLAWHPIGYILCSGSNDHTTKFWCRNRPGDPARDKFNV
GHSQGYGDQNSALAGRMPGNFPGTEPPTTPGPFAAGLTRNEGNIPGVGVAMPFSIPSFDPSAQGEQRP
PPLASMPLGAPPLPPGPHPSLLAANQQQPYQQQNPQQIQHHQAHPQQMPQLPLPPSNLPQLQPPSHL
SMLSHPHLPRNPPQLSPLGMPSSMPGPMPTSLPMPMPSQMGMPGALNQMVPPFQQGHFMGMNP
MHSGAAPQVGGIANGLPNMQGPSNASGNQMYQSAGAFNRPQAGPMPPMPGHNPYQPGNPNAAG
MPSNFNLPSGMPPPLPPGPPPHG

WDL2

MCKSDTCHAPKSHISASWADTGHVQVWDFSSHLNALAESEARVSQEDSPVTNQAPLVKFGGHKDEGYAI
DWSPIVAGRLVSGDCKNCIYLWEPTSGTTWNTSTNPFVGHSASVEDLQWSPTEADVFASCSVDGNIAIW
DIRLGNSPAAKIKAHDADVNVISWNRLASCMLASGSDDGTFSIRDLRLLKDGDSVVAHFEYHKHPITSIEW
SPHEASTLAVSSSDNQLTVWDLSLERDEEEEAEFKAKTQEQVNAPTDIPPQLLFVHQGQKDLKELHWHSQ
IPGMVISTAADGFNILMPSNIETALPPSNDVQMS

WDL3

MQASLNNGLHNFGRIELSNLPLPARIWTIGDGPCSSGMQPGPASVVLKHCKGRTNEKCKDVTTLDWNGE
GTLLATGSYDGQARIWNTDGELMTTLNKHKGPIFSLKWNKKGDYLLSGSFDETAIVWDIKTGEWKQQFEF
HSAPTLDVDWRTNVSFATCSTDHMIYVCKVGENRPVKTFSGHQGEVNAIKWDPTGSLLASCSDDSTAKI
WSMKQDTYVHDLKEHSKEIYTIRWSPTGTGTNNPNQQLVLASASFDSTIKLWDVESGGVLHSLKGHRDSV
YSVAFSPNGEYLASGSLDRCLHIWSVKQGKLVKTYTGNGGIFEVCWNKEGDKVAACFSNEVVCVFDFRL

WDL4	MMYGDPQQQQQQQQQQLHPQQQQQQLQQHLQSHQQHPQPPQHHPQFGEFPRGPQQQQQQQQ<br>QQQPPPPPPPMMRQSSALSSNMGGQPDYHHQPLAQPPHPPYDAPGDSFAAKRIRKIGQRRAVDYTSTV<br>VRYLQIRMFQRDSRDRTVLQPTPAAAIDMLPAVAYSDNPSTSFASKFVHTSLNKNRCSINRVLWTPSGRRLI<br>TGSQSGEFTLWNGQSFNFEMILQAHDQAVRSMVWSYNENWMVTGDDGGSIKYWQNNMNNVKAYK<br>SAHKESVRDLSFCRTDLKFCSCSDDTTVKVWDFARCQEERSLSGHGWDVKTVDWHPTKSLLASGGKDNL<br>VKLWDAKSGRELSSFHGHKNTVLCVKWNQNGNWVLTASKDQIIKLYDIRAMKELESFRGHQKDVTALAW<br>HPFHEEYFVSGSLDGSIFHWLVGHDTPQVENSQCP
WDL5	MTMEGSSESGWQKSDSSRGFNASAVSDRNPRLFRGSGIRSSGDASQESGSIPVRKERDRVVLAHRDLHR<br>TPAGLSGVCEDDVERGPVVRAIEWNDVSLRQWLDDPERSVDAIECLHIFTQIVDIVNLAHSQGVVVNNVR<br>PSCFVMSSFHRVSFIESASCSSESGSDSMEDMSNGQTAEFKGSSSPLPHDSHQHRSHFHTEDIRLERNQTN<br>ASHLASETSCLQSSSVYPMPKSLEVGSNVKQAEEKKHSFPMKQILLMETNWYTSPEEAAGAPSSCASDVY<br>RLGVLLFELFCTFSSAEEKSSTMSSLRHRVLPPQLLLKWPKEASFCLWLLHPDASGRPKMSELLQSEFLN<br>EPREDIEEREVAIELRERIDEQELLLEFLLMMQQRKQEAADNLQDSISFLSSDIEEVTKQQSLLWRRGGSCPE<br>LGKDSASSLPSVKVVDDGDSSCLASRKRYRPGLQNQNEEELGDGVHNCENPEEPAENQGSTLSKSSRLIKN<br>FKKLESAYFLTRRRSIKSTGKPLTRQSPISSDGRRSIVATERSSINNLPSVNRFSEGRQSGWVNSFLEGLCKYLS<br>FSKLKVRADLKQGDLLNSSNLVCSLSFDRDGEFFATAGVNKKIKVFEYATVLNEDRDIHYPVVELSSRSKLSSI<br>CWNSYIKSQIASSNFEGVVQVWDVTRSQVFLEMREHERRVWSVDFSLADPTMLASGSDDGSVKLWNIN<br>QGASVGTIKTKANVCCVQFPADSSRSLAFGSADHRIYYYDLRNSKMPLCTLIGHEKTVSYVKFIDSANLVSA<br>STDNTLKLWDLSMCTSRVLDSPLQSFTGHTNVKNFVGLSVSEGYIATGSETNEIFIYHKAFPMPALAFKFGS<br>TDPLSGDEVDDNSQFISSLCWRGQSSTLVAANSMGNIKLLEMV
WDL6	MTMEGSSESGWQKSDSSRGFNASAVSDRNPRLFRGSGIRSSGDASQESGSIPVRKERDRVVLAHRDLHR<br>TPAGLSGVCEDDVERGPVVRAIEWNDVSLRQWLDDPERSVDAIECLHIFTQIVDIVNLAHSQGVVVNNVR<br>PSCFVMSSFHRVSFIESASCSSESGSDSMEDMSNGQTAEFKGSSSPLPHDSHQHRSHFHTEDIRLERNQTN<br>ASHLASETSCLQSSSVYPMPKSLEVGSNVKQAEEKKHSFPMKQILLMETNWYTSPEEAAGAPSSCASDVY<br>RLGVLLFELFCTFSSAEEKSSTMSSLRHRVLPPQLLLKWPKEASFCLWLLHPDASGRPKMSELLQSEFLNEPR<br>EDIEEREVAIELRERIDEQELLLEFLLMMQQRKQEAADNLQDSISFLSSDIEEVTKQQSLLWRRGGSCPELGK<br>DSASSLPSVKVVDDGDSSCLASRKRYRPGLQNQNEEELGDGVHNCENPEEPAENQGSTLSKSSRLIKNFKK<br>LESAYFLTRRRSIKSTGKPLTRQSPISSDGRRSIVATERSSINNLPSVNRFSEGRQSGWVNSFLEGLCKYLSFSK<br>LKVRADLKQGDLLNSSNLVCSLSFDRDGEFFATAGVNKKIKVFEYATVLNEDRDIHYPVVELSSRSKLSSICW<br>NSYIKSQIASSNFEGVVQVWDVTRSQVFLEMREHERRVWSVDFSLADPTMLASGSDDGSVKLWNINQG<br>ASVGTIKTKANVCCVQFPADSSRSLAFGSADHRIYYYDLRNSKMPLCTLIGHEKTVSYVKFIDSANLVSASTD<br>NTLKLWDLSMCTSRVLDSPLQSFTGHTNVKNFVGLSVSEGYIATGSETNEIFIYHKAFPMPALAFKFGSTDP<br>LSGDEVDDNSQFISSLCWRGQSSTLVAANSMGNIKLLEMV

Table S4: The primers used for qRT-PCR

Gene	Forward primer	Reverse primer
MYBL1	GACTGATTACGTTAAGCTTCATG	CAGCTATTAGCGACCATCTGTTG
MYBL3	ACTACAAGAGGAGGATCTCAT	CGTGGTAAACCCAATGGTGAT
MYBL5	ACTGCAACCCTAACGATTATTC	ACGTCGAACATCTGGGCGTAG
MYBL6	ATGTGAAGCCAAAGCTGAGGA	ACTGAGTATGAGATCCTCCTC
MYBL7	GACCCATTCATAGCTTCCTCATC	ATCTTCCACGTTTAAGCACGGAAC
bHLHL1	GTGCCCTCAACAAGGGATCTTAG	TAGAACCACTCGGACTCCGT
bHLHL4	GAGATGTTCTATCTTACATCA	GCCAATCTCCACTACTCCTGAT
WDL2	GTATGGGACTTCAGCTCTCATC	ATTCCATGTTGTACCAGACGTA
WDL6	ATGCTTCTGCCGTCTCGGAT	CTGTCTCAAGCTAACATCAT
GADPH	GGTTATCAATGATAGGTTTGGCA	CAGTCCTTGCTTGATGGACC

Table S5: The potential VcMYBL1, VcbHLHL1 and VcWDL2 protein interactions were predicted using STRING software

Query sequence	STRING protein	Annotation	Identity	Bitscore
	Vitis vinifera			
MYBL1	VIT_11s0016g01300.t01	Putative uncharacterized protein	55%	278
bHLHL1	MYC1	Putative uncharacterized protein	73%	949
WDL2	VIT_11s0016g02270.t01	Putative uncharacterized protein	85%	521
	Arabidopsis thaliana			
MYBL1	MYB12	Myb domain protein 12; Transcription factor that activates the expression of CHS, CHI, F3H and FLS1. Flavonol-specific activator of flavonoid biosynthesis	61%	193
bHLHL1	TT8	TRANSPARENT TESTA 8; Transcription activator, when associated with MYB75/PAP1 or MYB90/PAP2. Involved in the control of flavonoid pigmentation. Plays a key role in regulating leucoanthocyanidin reductase (BANYULS) and dihydroflavonol-4-reductase (DFR). Not required for leucoanthocyanidin dioxygenase (LDOX) expression	73%	332
WDL2	HOS15	WD-40 repeat family protein	75%	528

* Query sequence: Input protein sequence; STRING protein: Database protein sequence; Annotation: Database annotation; Identity: Proportion of identical parts; Bitscore: Similarity score.
